# Magnetic space groups *versus* representation analysis in the investigation of magnetic structures: the happy end of a strained relationship

**DOI:** 10.1107/S2052520624007479

**Published:** 2024-09-10

**Authors:** J. Rodriguez-Carvajal, J. M. Perez-Mato

**Affiliations:** a Diffraction Group Institut Laue-Langevin 38054Grenoble Cedex 9 France; b Facultad de Ciencia y Tecnología Universidad del País Vasco UPV/EHU, Apartado 644 48080Bilbao Spain; Oak Ridge National Laboratory, USA

**Keywords:** magnetic space groups, representation analysis, magnetic structures, irreducible representations, mode decomposition

## Abstract

In recent decades, sustained theoretical and software developments have clearly established that representation analysis and magnetic symmetry groups are complementary concepts that should be used together in the investigation and description of magnetic structures. Historically, they were considered alternative approaches, but currently, magnetic space groups and magnetic superspace groups can be routinely used together with representation analysis, aided by state-of-the-art software tools. After exploring the historical antagonism between these two approaches, we emphasize the significant advancements made in understanding and formally describing magnetic structures by embracing their combined use.

## Abstract 2

After reviewing the historical unjustified antagonism between the application of magnetic symmetry groups and representation analysis in the investigation of magnetic structures, we highlight the progress achieved in recent years by moving beyond this entrenched divide and applying both concepts together in a complementary manner.

## 1. Introduction

Historically, two approaches for describing long-range magnetic ordering in a crystal were developed in parallel. The first approach involves describing the magnetic arrangement under the symmetry constraints of a specific magnetic space group (MSG) in the case of a commensurate structure or a magnetic superspace group (MSSG) if the structure is incommensurate. The second approach, called representation analysis (RA), considers one or more irreducible representations (irreps) of the space group of the paramagnetic phase and their corresponding spin basis vectors, to build up the arrangement of atomic magnetic moments in the structure. For decades, these two approaches were considered mutually exclusive descriptions, largely due to an unfortunate personal controversy at the early stages of their development. However, both descriptions are complementary parts within a unique framework, namely the description of a system where a symmetry-breaking process has occurred. The perception began to change with the availability of digital databases of MSGs and MSSGs in the *Isotropy Software Suite* (Stokes & Campbell, 2010 ▸) and the extension to magnetic structures of software tools using together symmetry groups and irreps, which were originally developed for the analysis of structurally distorted structures. This was followed by several papers (Petříček *et al.*, 2010 ▸; Rodríguez-Carvajal & Bourée, 2012 ▸; Perez-Mato *et al.*, 2012 ▸, 2015 ▸; Damay, 2015 ▸; Rodríguez-Carvajal & Villain, 2019 ▸), which discussed the relationship between the two concepts, stressing their complementarity. The aim of this paper is to review these historical developments, without entering into the theoretical formalism, and show how the two concepts work together when state-of-the-art software tools, which combine irreps and magnetic symmetry groups, are used.

## 2. History of a strained relationship between RA and MSGs

In the 1960s and 1970s, with the development of neutron sources and instrumentation, the experimental determination of magnetic structures started to grow. While MSGs had been developed in detail from the mathematical viewpoint [see, for instance, Opechowski & Guccione (1965 ▸)], no systematic practical method existed for enumerating possible MSGs for some given diffraction data, leaving MSG theory far from practical applications. At best, once a magnetic structure was determined by whatever method, its MSG was identified and reported. In contrast, the RA method, developed by E. F. Bertaut and co-workers, offered a systematic approach for the enumeration and description of possible spin arrangements consistent with experiment. The RA method was applied to the studies of commensurate magnetic structures and incommensurate helical magnetic structures; see for instance the references cited by Bertaut (1963 ▸, 1968 ▸).

The RA method proposes that spin arrangements can be classified based on their transformation properties under the operations of the space group (SG) of the paramagnetic phase, rather than their invariance properties as done with magnetic symmetry groups. These transformation properties can be described by irreducible representations (irreps) of the SG. By considering the wavevector(s) of the magnetic ordering [propagation vector(s)], which are directly obtainable from diffraction data, the possible relevant irreps can be significantly narrowed down to those corresponding to the observed wavevector(s). For each of these irreps, standard representation group theory is then applied to construct a basis of irrep-adapted spin modes (spin basis vectors). These basis vectors or modes can be used to describe any spin arrangement that satisfies the transformation properties of the irrep. In many cases, magnetic ordering conforms to a single irrep, though complex scenarios may require several irreps. This characteristic makes the method highly efficient and successful in systematically enumerating and describing possible spin arrangements.

It is noteworthy that although the RA method is closely related to the Landau theory of symmetry-breaking phase transitions, the two extensive articles by Bertaut (1968 ▸, 1971 ▸), where the method was thoroughly reviewed, do not mention Landau theory. This is surprising, especially considering the emphasis on the suitability of irrep-adapted variables for diagonalizing the Hamiltonian of the system. In fact, at this time, based on Landau theory, RA and ordinary space groups were being used together to study structural phase transitions and characterize structurally distorted structures [see for instance Dvořák (1971 ▸), Dvořák & Petzelt (1971 ▸) and references therein]. Irreps were utilized to explain or to predict the space group of the distorted structure and to describe the observed atomic displacements, so that symmetry groups and irreps were considered together within a common framework. In contrast, in the case of magnetic structures, after Bertaut’s publication in 1968, intense discussion arose regarding the capabilities of RA *versus* MSG analysis for the description or ‘classification’ of magnetic structures, treating them as alternative approaches. In the case of commensurate structures, early claims by Bertaut (1968 ▸) that MSGs were inadequate for dealing with certain commensurate magnetic structures were refuted in a formal mathematical manner by Opechowski & Dreyfus (1971 ▸). They demonstrated that any commensurate magnetic structure can indeed be assigned an MSG, and any additional correlations observed in the structure not dictated by the MSG could be manually added. The discussion was also extended to incommensurate magnetic structures, where a ‘symmetry invariance’ approach was in fact not possible at that time, as MSSGs were not formally established until 1980 (Janner & Janssen, 1980 ▸). Despite the obvious advantage of RA being applicable for both commensurate and incommensurate propagation vectors without significant difference, Opechowski & Dreyfus (1971 ▸) insisted on the use of MSGs as an alternative to the RA description, provided that some ‘algebraic’ relations were added.

Participants in this debate eventually reached a tentative agreement that the two classification schemes were ‘equivalent’. However, this mutual acceptance of equivalence most likely added more confusion, because defining the symmetry group of a system, *i.e.* its invariance symmetry operations, is generally different from defining its transformation properties for the operations of a group that is not its symmetry group. In essence, the discussion was misdirected, because the crucial question was not how to assign a label or a classification stamp to an already known magnetic structure, but rather how to develop a systematic method for constructing all possible magnetic arrangements that could fit some given diffraction data. In this regard, the proposed RA method was undoubtedly the most efficient method available at that time. Full listings of irreps of space groups were available (Kovalev, 1965 ▸; Bradley & Cracknell, 1972 ▸), and using standard group-theoretical methods, the construction of irrep spin basis vectors to build up the spin arrangements was feasible. In contrast, the theory of MSGs remained detached from practical applications for many years. As stated by Opechowski & Guccione (1965 ▸): ‘It is not our intention here to formulate practical rules for determining the characteristics …. of an invariant spin arrangement from some given experimental data …’. Unfortunately, this lack of ‘practical rules’ persisted for many years, perhaps exacerbated by the assumed equivalence between the RA method and MSGs. If there was already a practical method available — the RA method — and both approaches were deemed equivalent, the development of practical rules for MSGs seemed unnecessary. Consequently, the RA method became more widely applied, and in the case of commensurate structures, the MSGs were often not identified or mentioned.

By the 1990s, the program *KAREP* (Hovestreydt *et al.*, 1992 ▸) and the *ISOTROPY* program (Stokes, 1995 ▸) were released, offering the capability to generate space group irreps. The *KAREP* program was freely available and was subsequently utilized by the *FullProf Suite* (Rodríguez-Carvajal, 1993 ▸) to automate the generation of irrep basis vectors. Over the following years, additional software tools serving the same purpose emerged, such as *MODY* (Sikora *et al.*, 2004 ▸) and *SARAh* (Wills, 2000 ▸). Consequently, by the turn of the century, the RA method was supported by software tools for its automatic application. As a consequence, it became prevalent in almost all published works, both for determining and describing magnetic structures.

During this period, the work of Yu. A. Izyumov and his co-authors also played a significant role in establishing the prevalence of the RA method and the general devaluation of MSGs. Through the publication of several papers where the RA method was thoroughly applied to various experimental cases, they contributed to its widespread adoption. In 1981, they published a book in Russian titled ‘Neutron diffraction of magnetic materials,’ which was later translated into English with considerable success (Izyumov *et al.*, 1991 ▸). In this work, the two concepts — RA and MSGs — were again compared as alternative approaches. Unlike previous discussions, this comparison did not lead to an equivalence conclusion; rather, the RA method was deemed highly preferable. The book in fact includes a subchapter titled ‘Insufficiency of the Description of the Symmetry of Magnetic Bodies with the Aid of Shubnikov Groups.’, and for commensurate structures, this ‘insufficiency’ was illustrated with two examples. One example was a ferromagnetic ordering within a cubic structure, where the spins are oriented along one of the main crystallographic axes. In this case, the magnetic ordering within the structure results in a reduction of its symmetry from cubic to tetragonal, with its MSG being necessarily a tetragonal group. The perceived insufficiency in this scenario stemmed from the fact that, within experimental resolution, the structure appeared to remain cubic. Consequently, it was argued that the MSG was not acceptable as the symmetry group of the entire system. As emphasized by the authors themselves, who reproduced a Landau citation, this reasoning contradicts the accepted understanding, including that of Landau, that the MSG describes the symmetry of the entire system, taking into account that the presence or absence of time reversal in the group operations is irrelevant for non-magnetic degrees of freedom. In reality, there is no *insufficiency* of the MSG in this case. The MSG fulfils its purpose, which is to describe the symmetry constraints of all degrees of freedom that are maintained *exactly* in this ferromagnetic phase. This holds true regardless of the magnitude of couplings among these degrees of freedom in the Hamiltonian or free energy of the system. The absence of an observable tetragonal structural distortion accompanying the ferromagnetic order can be attributed to the smallness of magnetostriction, stemming from the weakness of spin-orbit coupling. However, this coupling, no matter how small, is symmetry allowed and will be present. The MSG inherently considers this and cannot impose any constraint that may be released due to this coupling. It is important to note that if one were to accept the argument presented in this book that the MSG does not represent the symmetry of the system as a whole, then it would be challenging to explain phenomena such as the magnetically induced ferroelectricity present in commensurate multiferroics of type II. In these cases, the magnetic ordering reduces the symmetry to a polar MSG, enabling spontaneous electric polarization. Moreover, similar to the example of the ferromagnetic cubic structure discussed by Izyumov *et al.* (1991 ▸), the structural distortion causing the small macroscopic electric polarization observed in these systems is typically too subtle to be detected by conventional diffraction techniques.

The second example provided in the mentioned book to illustrate the ‘insufficiency’ of the MSGs was the magnetic structure of CrCl_2_. The space group of the paramagnetic structure of this compound is *Pnnm*, with a Cr atom at the origin and a symmetry related one at (½, ½, ½). The reported magnetic structure for this compound is collinear, with propagation vector (0, ½, ½). According to the published model, the two mentioned Cr atoms in the parent unit cell possess opposite moments (*u*, *v*, *w*) and (−*u*, −*v*, −*w*) along a general direction. The MSG of such magnetic arrangement is however very low, namely triclinic 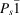 [Belov–Neronova–Smirnova (BNS notation)], with the two Cr sites being symmetry independent under this MSG. Consequently, the reported correlation between the two moments cannot be explained by the MSG. This type of scenario where the spin arrangement exhibits correlations not dictated by the MSG, had already been discussed in prior literature (Opechowski & Dreyfus, 1971 ▸). In those discussions, authors did not hesitate to incorporate manually these additional relations to establish a ‘classification label’ for the structure. However, in Izyumov *et al.* (1991 ▸), this scenario was revisited with the CrCl_2_ example to emphasize that if the MSG fails to include the observed correlation between the spins, then there might be something lacking in the MSG approach. Again, here there is no *insufficiency* with the MSG, as it performs its designated function by defining only correlations that are *protected by symmetry*.

It is worth noting that this second example might not have been the most suitable choice. In this specific case, neither the RA approach nor the MSG can explain the reported correlation between the two spins. The magnetic representation of the Cr spins for the observed propagation vector includes only one two-dimensional irrep, indicating that the spin basis vectors for this single irrep encompass all possible spin arrangements for this propagation vector. This means that the RA assigning a single irrep to the spin arrangement does not bring in this case any constraint to the spins and cannot explain either the reported correlation. But many structures exhibit spin correlations that are indeed not entirely prescribed by MSGs, and some of these correlations can be elucidated by the fact that the spin arrangement corresponds to some specific irrep(s). As discussed above, this occurs because MSGs only define symmetry-protected constraints. If the constraints imposed by the MSG do not fully align with those of the active irrep(s), it means that the model allows for additional spin degrees of freedom corresponding to other irreps, which may be released through symmetry-allowed couplings in the Hamiltonian or free energy of the system. Whether these additional degrees of freedom are observed in the experimental spin arrangement depends on the magnitude of these couplings. Nevertheless, by definition, MSGs must incorporate them in any case.

If one considers dominating isotropic Heisenberg interactions between the spins (absence of spin-orbit coupling), it may be possible to have additional symmetries in the Hamiltonian. This may be the case of CrCl_2_ in which the observed correlation, (*u,v*, *w*) and (−*u*, −*v*, −*w*) along a general direction, may be the consequence of a strong isotropic exchange between the spins of the two sites. However, if all potential spin couplings allowed by the Hamiltonian or free energy under the *Pnnm* symmetry are considered including spin-orbit coupling, the observed correlation between the spins may disappear or the arbitrary direction of the spins may become blocked to a specific crystallographic direction. In fact, there is currently an intensive revival of research about the approximate additional symmetries that can be associated with a magnetic structure, when spin-orbit coupling is neglected. This is the subject of the so-called *spin space groups*, which were introduced in the 1960s–1970s (Brinkman & Elliot, 1966 ▸, Litvin & Opechowski, 1974 ▸). They are now mainly used to explain specific electronic band features (Liu *et al.*, 2022 ▸; Šmejkal *et al.*, 2022 ▸), but they can also provide new insights into the spin correlations observed in magnetic structures that cannot be explained by their MSG and/or some irrep. This is an interesting subject beyond MSG and RA, which will need to be thoroughly investigated in the future.

To summarize, by the end of the 20th century, the RA method had become the predominant approach for analysing and determining magnetic structures, both commensurate and incommensurate. This was largely facilitated by the availability of software tools that enabled automatic application of the RA method. In contrast, the different role played by the magnetic symmetry groups in the characterization of these structures was generally overlooked, and consequently, magnetic symmetry groups appeared to be undervalued. This perception began to change in the following decade.

## 3. How RA and MSGs work together

### 3.1. General considerations

As previously mentioned, RA and space group symmetry began to be applied in the analysis of structurally distorted structures in the 1970s, not as competing approaches, but as complementary concepts. Following Landau theory, a structurally distorted structure relative to a parent structure of higher symmetry (real or virtual) can be viewed as the outcome of a symmetry-breaking instability. In this scenario, one or more primary order parameters (unstable distortion modes) can be defined, transforming according to one or more irreps of the parent space group. The irrep of an order parameter specifies its transformation properties for all operations of the parent space group. Some of these operations will maintain the order parameter invariant and consequently, the corresponding distortion. These operations define the space group symmetry remaining with the presence of the distortion. In the case of multi-dimensional order parameters (*i.e.* multi-dimensional irreps), this space group will generally vary based on the direction taken by the order parameter, *i.e.* the combination of the different unstable modes forming the observed distortion.

Similarly, magnetic ordering is a symmetry breaking process involving the loss of time-reversal symmetry. Time reversal, which macroscopically reduces to switching the direction of all average magnetic moments in the structure is necessarily a symmetry operation of any paramagnetic structure, where these moments are necessarily zero, while it is necessarily absent as symmetry operation in any magnetically ordered structure. A magnetic structure can therefore be conceptualized as a distorted structure of lower symmetry compared to the paramagnetic structure. Consequently, one or more order parameters responsible for the magnetic order can be defined, which transform according to irreps of the parent symmetry group, including time reversal, *i.e.* of the grey MSG associated with the ordinary parent space group. The relevant irreps are necessarily odd for this operation, since the magnetic order breaks it, being trivially related one to one to the irreps of the ordinary parent space group. The possible MSGs are then determined by the operations that, according to the irrep(s), keep the order parameter(s) invariant. In cases involving multi-dimensional irreps, the resulting MSG typically varies depending on the direction of the order parameter, *i.e.* on the specific combination of the corresponding spin basis vectors.

As highlighted in the previous section, irreps and MSGs serve distinct purposes in the characterization of a magnetically ordered phase. irreps delineate the transformation properties of spin configurations that may be unstable within the system’s Hamiltonian or free energy. On the other hand, MSGs define the invariant symmetry of the realized spin arrangement, representing the symmetry that persists in the distorted phase. The constraints outlined by the MSG extend beyond just the symmetry-breaking spin ordering; they encompass all degrees of freedom, including both structural and magnetic aspects, which may emerge in the distorted phase through various couplings. The difference and complementarity of these two concepts become evident in scenarios involving multi-dimensional irreps. In such cases, different MSGs can manifest depending on the direction taken by the order parameter. The possible MSGs corresponding to a given irrep of dimension greater than one are referred to as isotropy subgroups. The selection of an arbitrary direction of the order parameter yields the lowest symmetry group, which is referred to as the kernel of the irrep. Special directions of the order parameter correspond to higher symmetry subgroups that are called the epikernels of the irrep.

Consequently, spin basis vectors associated with a single multi-dimensional irrep, depending on how they are combined, can describe magnetic arrangements exhibiting different MSGs, thereby generally possessing distinct physical properties. For example, there is a crucial distinction between stating that a magnetic structure corresponds to the two-dimensional irrep mX1 (CMDL notation) of the parent space group *Pnmaversus* stating that the MSG of this magnetic structure is *P_b_mn*2_1_ (No 31.129) (BNS notation), with its standard setting related with that of the parent space group *Pnma* by the basis transformation (−**b**, 2**a**, **c**; ¾, ¼, 0). The MSG not only specifies the symmetry invariance constraints of the spin arrangement but also encompasses the constraints of all degrees of freedom. The *P_b_mn*2_1_ MSG indicates that the centrosymmetry of the paramagnetic phase has been lost, resulting in a polar structure along the *c* axis of the *Pnma* setting. This loss of centrosymmetry allows, if the material is an insulator, for the manifestation of magnetically induced electric polarization along this direction. The spin arrangement within this structure can indeed be constructed by spin basis vectors corresponding to the irrep mX1. However, altering the combination of these basis vectors can yield different MSGs. For instance, if the basis vectors are combined differently, the MSG might become the centrosymmetric monoclinic MSG *P_a_*2_1_/*m* with its standard setting related to that of the parent *Pnma* by the transformation (2**a**, **b**, **c**; 0, 0, 0), which constitutes another epikernel of the same irrep. This MSG, with its monoclinic axis aligned along the parent *b* axis, is then an alternative centrosymmetric symmetry of a magnetic ordering according the irrep mX1. Furthermore, for an arbitrary combination of the basis vectors, the resulting symmetry might be even lower. In essence, a magnetic structure described by the irrep mX1 of the space group *Pnma* can exhibit various symmetries. If it aligns with one of the two epikernels, additional symmetry protected spin correlations implied by this MSG must be considered. Therefore, while the irrep delineates the transformation properties of the spin configuration, the MSG provides a comprehensive description of the structural and magnetic constraints imposed on the system in its ordered state.

The combined application of irreps and symmetry groups in the examination of structurally distorted structures gained traction in the early 1970s, coinciding with the availability of systematic lists of irreps for space groups. Structural phase transitions were expected to involve a single order parameter, *i.e.* a single active irrep. Consequently, one could determine the most probable symmetry groups for the resulting low-symmetry structures by manually computing the isotropy subgroups for each irrep. For example, publications such as Aleksandrov (1976 ▸) and Perez-Mato *et al.* (1981 ▸) provided systematic derivations of possible symmetries for distorted phases according to a single irrep for a given parent space group.

### 3.2. Development of databases and computer tools

#### 3.2.1. Structural distortions

By 1981, Hatch and Stokes initiated the compilation of a catalogue featuring computer-calculated isotropy subgroups for all irreps of all space groups at special points of symmetry in the first Brillouin zone. This endeavour culminated in the publication of a comprehensive book (Stokes & Hatch, 1988 ▸), which served as a valuable resource for researchers. Conversely, many publications identified the irrep associated with the order parameter responsible for the transition when the space group of the distorted phase was known. This enabled the characterization of the specific mode or modes that were unstable in the parent structure, thus facilitating a deeper understanding of the transition process. In the early 2000s, the advent of computational tools enabled the comprehensive analysis of structurally distorted structures through RA. A notable development was the release of the *ISODISPLACE* program (Campbell *et al.*, 2006 ▸), later renamed *ISODISTORT*, as part of the *ISOTROPY Software Suite* (Stokes *et al.*, 1995 ▸). This online tool facilitated the determination of possible isotropy subgroups for any irrep of a given parent space group, or the possible symmetries in the case of several irreps being involved. This allowed researchers to generate starting models for structurally distorted low-symmetry structures, to be used for refinement of experimental diffraction data. *ISODISTORT* was also able to decompose experimental distortions into irrep modes when both parent and distorted structures were known. The program provided a set of basis modes for all relevant irreps, along with the amplitudes of these modes in the experimental structure. Subsequently, the Bilbao Crystallographic Server collaborated with the *ISOTROPY* team to develop *AMPLIMODES* (Orobengoa *et al.*, 2009 ▸; Perez-Mato *et al.*, 2010 ▸), a program for the irrep mode decomposition of commensurate displacive distorted structures. The integration of this irrep mode decomposition into the *FullProf Suite* (Rodríguez-Carvajal, 1993 ▸) and the *JANA* (Petříček *et al.*, 2014 ▸, 2023 ▸) programs, further streamlined the refinement process. With the space group of the distorted structure established or assumed, *FullProf* or *JANA* could refine the amplitudes of irrep modes provided by *ISODISTORT* or *AMPLIMODES* instead of refining individual atomic positions. In this approach to structure refinement, akin to the traditional RA method for magnetic structures, the bases of irrep modes are constrained by the space group for the distorted structure. This convergence of computational tools significantly advanced the analysis and refinement of structurally distorted materials.

#### 3.2.2. Commensurate magnetic ordering

A complete compilation of MSGs was published by Litvin in the form of a freely available huge electronic book (PDF format), which mimics the *International Tables of Crystallography* (Litvin, 2001 ▸, 2008 ▸, 2013 ▸). The 2010s were also marked by a significant progress in the extension to magnetic structures of tools and methods originally developed for the analysis of structurally distorted structures. The *ISOTROPY Software Suite* played a pivotal role by providing digital listings of all MSGs in June 2010, followed by the integration of magnetic structure analysis into *ISODISTORT* in August of the same year. The Bilbao Crystallographic Server also contributed to this effort by releasing online MSG listings in a user-friendly format, and developing practical tools that employ them (Gallego *et al.*, 2012 ▸). In addition, during this period refinement programs like *JANA* were extended to accommodate magnetic structures. The approach in *JANA* involves enumerating and classifying possible irreps for observed propagation vectors, calculating isotropy subgroups for each irrep, and generating magnetic structure models under each potential MSG for refinement against experimental data. Simultaneously, enhancements were made to *FullProf* to support MSGs for magnetic structures. Both *FullProf* and *JANA* also extended to magnetic structures the option that they already had for structural distortions, and enabled refinements with a parameterization in terms of separate irrep modes within a specified MSG, applying the irrep decomposition that the program *ISODISTORT* can provide.

The development of the International Union of Crystallography’s magCIF format for communicating magnetic structures has been crucial for all these developments, as it has facilitated universal portability among programs. This format allows the investigation and determination of magnetic structures through automated RA and magnetic symmetry groups, supported by various resources for analysis, refinement, and visualization — all adhering to the magCIF standard. Consequently, researchers could explore magnetic structures more efficiently, combining RA and magnetic symmetry groups in a seamless manner. The description of the magCIF format is provided in the following link: https://www.iucr.org/__data/iucr/cifdic_html/3/MAGNETIC_CIF/index.html.

#### 3.2.3. Incommensurate magnetic ordering

The emergence of MSSGs represents a significant development in understanding and characterizing incommensurate magnetic structures. Initially proposed in 1980 for incommensurate phases (Janner & Janssen, 1980 ▸), superspace symmetry groups gained recognition for their applicability to incommensurate magnetic structures much later, around 2012. This delay in recognition was addressed by subsequent publications that fully developed the theory of MSSGs for magnetic systems (Petříček *et al.*, 2010 ▸; Perez-Mato *et al.*, 2012 ▸, Stokes & Campbell, 2022 ▸) and integrated them into mainstream resources like *FullProf*, *JANA* and *ISOTROPY Software Suite*. MSSGs introduce a new type of transformation — global phase shifts of the incommensurate modulation — alongside traditional transformations like translations, rotations, inversion, and time reversal, to form symmetry operations. These global phase shifts preserve the energy of the incommensurate structure, making them compatible with other energy-invariant transformations. Symmetry operations in a well defined group not only preserve system invariance but also satisfy a physical condition: they must be a subset of operations preserving the system’s energy (Hamiltonian or free energy). This ensures that the constraints imposed by the symmetry group are robust and independent of variations in system parameters, such as temperature or pressure in the case of the free energy (as long as no phase transition takes place).

In the case of incommensurate structures, MSSGs provide constraints not only for the primary irrep(s) associated with the observed propagation vector(s) but also for any higher harmonics in the modulation. Once an MSSG is assumed to model the structure, it governs the symmetry properties (irreps) of all modulation harmonics through coupling with the primary irrep(s). Free software tools like *ISODISTORT* or *JANA* can derive MSSGs for each irrep or superposition of irreps associated with the observed propagation vectors, allowing for comprehensive analysis and characterization of incommensurate magnetic structures.

## 4. Conclusion

The role of RA and magnetic symmetry groups in the description and characterization of magnetic structures is now well established, with an analogy to structurally distorted structures. Software tools originally developed for structurally distorted structures are now available for magnetic structures, enabling the simultaneous application of RA and magnetic symmetry groups. However, there is a significant qualitative difference in how these tools should be applied to magnetic structures. In structurally distorted structures, once the symmetry group is established or assumed, structure determination typically relies solely on the constraints of the symmetry group. This is because strong couplings among all degrees of freedom usually result in significantly independent values for all released structural degrees of freedom allowed by the symmetry group. In contrast, for magnetic structures, the orders of magnitude of coupling mechanisms can vary significantly, with some degrees of freedom remaining silent within the experimental resolution due to weak coupling to the primary ordering mechanism. These silent degrees of freedom, particularly in magnetic moments, may correspond to other irreps besides the primary ones. In such cases, a structure refinement parameterized with the amplitudes of modes for different irreps allowed by the magnetic symmetry group is beneficial, as silent irreps can be directly identified and nullified in a refinement.

Once an MSG or MSSG is assumed for the structure, mainstream software resources facilitate the refinement with the magnetic structure consistently described under that magnetic symmetry group, and if the MSG is compatible with more than one irrep, RA becomes necessary again to separate through an irrep decomposition the degrees of freedom corresponding to each compatible irrep.

## References

[bb1] Aleksandrov, K. S. (1976). *Sov. Phys. Crystallogr.* **21**, 133.

[bb2] Bertaut, E. F. (1963). *Magnetism*, edited by G. T. Rado & H. Suhl, Vol. 3, p. 150. New York: Academic Press.

[bb3] Bertaut, E. F. (1968). *Acta Cryst.* A**24**, 217–231.

[bb4] Bertaut, E. F. (1971). *J. Phys. Colloq.* **32**, C1-462–C1-470.

[bb5] Bradley, C. J. & Cracknell, A. P. (1972). *The Mathematical Theory of Symmetry in Solids*. Oxford: Clarendon Press.

[bb6] Brinkman, W. F. & Elliot, R. J. (1966). *Proc. R. Soc. Lond. A*, **294**, 343–358.

[bb7] Campbell, B. J., Stokes, H. T., Tanner, D. E. & Hatch, D. M. (2006). *J. Appl. Cryst.* **39**, 607–614.

[bb8] Damay, F. (2015). *J. Phys. D Appl. Phys.* **48**, 504005.

[bb9] Dvořák, V. (1971). *Phys. Status Solidi B*, **45**, 147–152.

[bb10] Dvořák, V. & Petzelt, J. (1971). *Czech. J. Phys.* **21**, 1141–1152.

[bb11] Gallego, S. V., Tasci, E. S., de la Flor, G., Perez-Mato, J. M. & Aroyo, M. I. (2012). *J. Appl. Cryst.* **45**, 1236–1247.

[bb12] Hovestreydt, E., Aroyo, M., Sattler, S. & Wondratschek, H. (1992). *J. Appl. Cryst.* **25**, 544.

[bb13] Izyumov, Y. A., Naish, V. E. & Ozerov, R. P. (1991). *Neutron Diffraction of Magnetic Materials*. (First edition in 1981 in Russian.) New York: Consultants Bureau.

[bb14] Janner, A. & Janssen, T. (1980). *Acta Cryst.* A**36**, 399–408.

[bb15] Kovalev, O. V. (1965).* Irreducible Representations of the Space Groups.* London: Gordon & Breach.

[bb16] Litvin, D. B. (2001). *Acta Cryst.* A**57**, 729–730. 10.1107/s0108767301006547 11679704

[bb17] Litvin, D. B. (2008). *Acta Cryst.* A**64**, 419–424. 10.1107/S010876730800768X 18421131

[bb18] Litvin, D. B. (2013). *Magnetic Group Tables. 1-, 2- and 3-Dimensional Magnetic Subperiodic Groups and Magnetic Space Groups*. Chester: International Union of Crystallography. Available from https://www.iucr.org/publications/iucr/magnetic-group-tables.

[bb19] Litvin, D. B. & Opechowski, W. (1974). *Physica*, **76**, 538–554.

[bb20] Liu, P., Li, J., Han, J., Wan, X. & Liu, Q. (2022). *Phys. Rev. X*, **12**, 021016.

[bb21] Opechowski, W. & Dreyfus, T. (1971). *Acta Cryst.* A**27**, 470–484.

[bb22] Opechowski, W. & Guccione, R. (1965). *Magnetism*, edited by G. T. Rado & H. Suhl, Vol. IIA, p. 105. New York: Academic Press.

[bb23] Orobengoa, D., Capillas, C., Aroyo, M. I. & Perez-Mato, J. M. (2009). *J. Appl. Cryst.* **42**, 820–833.

[bb24] Perez-Mato, J. M., Manes, J. L., Tello, M. J. & Zuniga, F. J. (1981). *J. Phys. C Solid State Phys.* **14**, 1121–1132.

[bb25] Perez-Mato, J. M., Gallego, S., Tasci, E., Elcoro, L., de la Flor, G. & Aroyo, M. (2015). *Annu. Rev. Mater. Res.* **45**, 217–248.

[bb26] Perez-Mato, J. M., Orobengoa, D. & Aroyo, M. I. (2010). *Acta Cryst.* A**66**, 558–590. 10.1107/S0108767310016247 20720321

[bb27] Perez-Mato, J. M., Ribeiro, J. L., Petricek, V. & Aroyo, M. I. (2012). *J. Phys. Condens. Matter*, **24**, 163201. 10.1088/0953-8984/24/16/163201 22447842

[bb28] Petříček, V., Dušek, M. & Palatinus, L. (2014). *Z. Kristallogr. Cryst. Mater.* **229**, 345–352.

[bb29] Petříček, V., Fuksa, J. & Dušek, M. (2010). *Acta Cryst.* A**66**, 649–655. 10.1107/S0108767310030527 20962373

[bb30] Petříček, V., Palatinus, L., Plášil, J. & Dušek, M. (2023). *Z. Kristallogr. Cryst. Mater.* **238**, 271–282.

[bb31] Rodríguez-Carvajal, J. (1993). *Physica B*, **192**, 55–69.

[bb32] Rodríguez-Carvajal, J. & Bourée, F. (2012). *EPJ Web Conf.* **22**, 00010.

[bb33] Rodríguez-Carvajal, J. & Villain, J. (2019). *C. R. Phys.* **20**, 770–802.

[bb34] Sikora, W., Białas, F. & Pytlik, L. (2004). *J. Appl. Cryst.* **37**, 1015–1019.

[bb35] Šmejkal, L., Sinova, J. & Jungwirth, T. (2022). *Phys. Rev. X*, **12**, 031042.

[bb36] Stokes, H. T. (1995). *Ferroelectrics*, **164**, 183–188.

[bb37] Stokes, H. T. & Campbell, B. J. (2010). *ISO-Mag: Table of Magnetic Space Groups, Isotropy Software Suite*. https://iso.byu.edu.

[bb38] Stokes, H. T. & Campbell, B. J. (2022). *Acta Cryst.* A**78**, 364–370. 10.1107/S2053273322003898 35781417

[bb39] Stokes, H. T. & Hatch, D. M. (1988). *Isotropy Subgroups of the 230 Crystallographic Space Groups.* Singapore: World Scientific.

[bb40] Stokes, H. T., Hatch, D. M. & Campbell, B. J. (1995). *ISOTROPY Software Suite*. https://stokes.byu.edu/iso/isotropy.php.

[bb41] Wills, A. (2000). *Physica B*, **276–278**, 680–681.

